# Chemical analysis of pottery reveals the transition from a maritime to a plant-based economy in pre-colonial coastal Brazil

**DOI:** 10.1038/s41598-023-42662-5

**Published:** 2023-10-05

**Authors:** Marjolein Admiraal, Andre C. Colonese, Rafael G. Milheira, Dione da Rocha Bandeira, Alexandro Demathe, Adriana M. Pereira dos Santos, Thiago Fossile, Helen M. Talbot, Manon Bondetti, Alexandre Lucquin, Javier Montalvo-Cabrera, Luciano Prates, Alejandro Serna, Oliver E. Craig

**Affiliations:** 1 Department of Archaeology, BioArCh, University of York, York, YO10 5DD UK; 2 Department of Prehistory and Institute of Environmental Science and Technology (ICTA), Universitat Autònoma de Barcelona, 08193 Bellaterra, Spain; 3 Department of Anthropology and Archaeology, Federal University of Pelotas, Coronel Alberto Rosa 154, Pelotas, RS 96010-160 Brazil; 4 Programa em Patrimônio Cultural e Sociedade, Universidade da Região de Joinville, Paulo Malschitzki, 10, Zona Industrial Norte, Joinville, SC 89219-710 Brazil; 5 Museu Arqueológico de Sambaqui de Joinville, Dona Francisca, 600, Joinville, 89201-220 Brazil; 6 Sapienza Arqueologia e Gestão do Patrimônio Arqueológico, Wenceslau Alves dos Santos, 1002, Tubarão, SC 88704-208 Brazil; 7 Grupo de Pesquisa em Educação Patrimonial e Arqueologia (GRUPEP), Universidade do Sul de Santa Catarina, Av. José Acácio Moreira, 787, Tubarão, SC 88704-900 Brazil; 8 División Arqueología, Facultad de Ciencias Naturales y Museo, Universidad Nacional de La Plata, 1900 La Plata, Argentina

**Keywords:** Lipids, Anthropology, Archaeology

## Abstract

Understanding long-term dynamics of past socio-ecological systems is essential for their future management. The southern Atlantic Forest coast of Brazil with its biodiverse littoral zone and artisanal fishing communities, is a priority for conservation. Traditional maritime knowledge is thought to have a deep-history and indeed, marine exploitation can be traced back to the middle Holocene. As part of one of South America’s largest diasporas, Guarani groups reached the southern Brazilian coast at around 1000 years ago. Their impact on the long-standing coastal economy is unknown, due to poor preservation of organic remains. Through the first organic residue study on Guarani pottery, we show that maize rather than aquatic foods was the most dominant product in pottery at this time. By developing a mixing model based on carbon isotope values of saturated and mono-unsaturated fatty acids we propose new criteria for the identification of maize, opening up avenues for future research. Our data confirms the importance of maize to the pre-colonial Guarani, even in a highly productive coastal environment. The Guarani occupation of this region marks a significant departure from previous socio-economic systems, potentially leading to loss of traditional knowledge and alleviating anthropogenic pressure, albeit temporarily, on the marine environment.

## Introduction

The Atlantic Forest coast of Brazil is one of South America’s most productive aquatic ecotones. It is a biodiversity hotspot and a global priority region for biological conservation and restoration^1^. Today, marine ecosystem services provide food and livelihood for thousands of people along the Brazilian coastline, notably to rural communities that for a long time relied on a combination of small-scale fishing and plant cultivation as part of their local ecological knowledge. Such indigenous knowledge plays a fundamental role in modern sustainable resource use and biological conservation and is critical to the food security of riverine and coastal fisheries in Brazil^2^. The deep historical roots of indigenous knowledge, and its increasing value in conservation and development agendas, require a good understanding of the origin, changing nature and modern legacy of indigenous knowledge and practices. Charting changes in marine resource exploitation throughout the occupation history of this region therefore provides an opportunity to document the heritage of Brazil’s Atlantic fisheries, and to assess their longer-term sustainability in response to different environmental and cultural drivers of change, both key to securing their future conservation. Longitudinal studies that extend beyond landing records are crucial for monitoring anthropogenic pressure on the marine environment.

Early occupation of the region is recorded from ca. 7000 cal BP when coastal settlers built massive shell mounds (Sambaquis). Archaeological and scientific analysis have shown that the Sambaqui builders had resilient, durable economies focused on coastal resource exploitation supplemented with wild and domesticated plant resources^3^. Sambaquis culture only began to diminish at around 2200 cal BP, a period corresponding to environmental changes and the dissolution of large groups into smaller social units^4^. This decline is punctuated by the introduction of ceramics of the Taquara-Itararé (or proto-Jê) cultural tradition at ca. 1200 cal BP from the Brazilian Highlands^5,6^. Whether this represents the reoccupation and replacement of Sambaqui culture by Taquara-Itararé people, or a more complex situation of assimilation remains a topic of debate^4^. Recent research has shown that the Taquara-Itararé ceramic users continued the exploitation of the rich marine environment^4,7^. Toso et al.^4^ observed some of the most extreme marine-focused diets ever recorded by bone stable isotope analysis in these ceramic sites, which included the exploitation of high trophic level prey species such as sharks and rays^8^.

**Figure 1:**
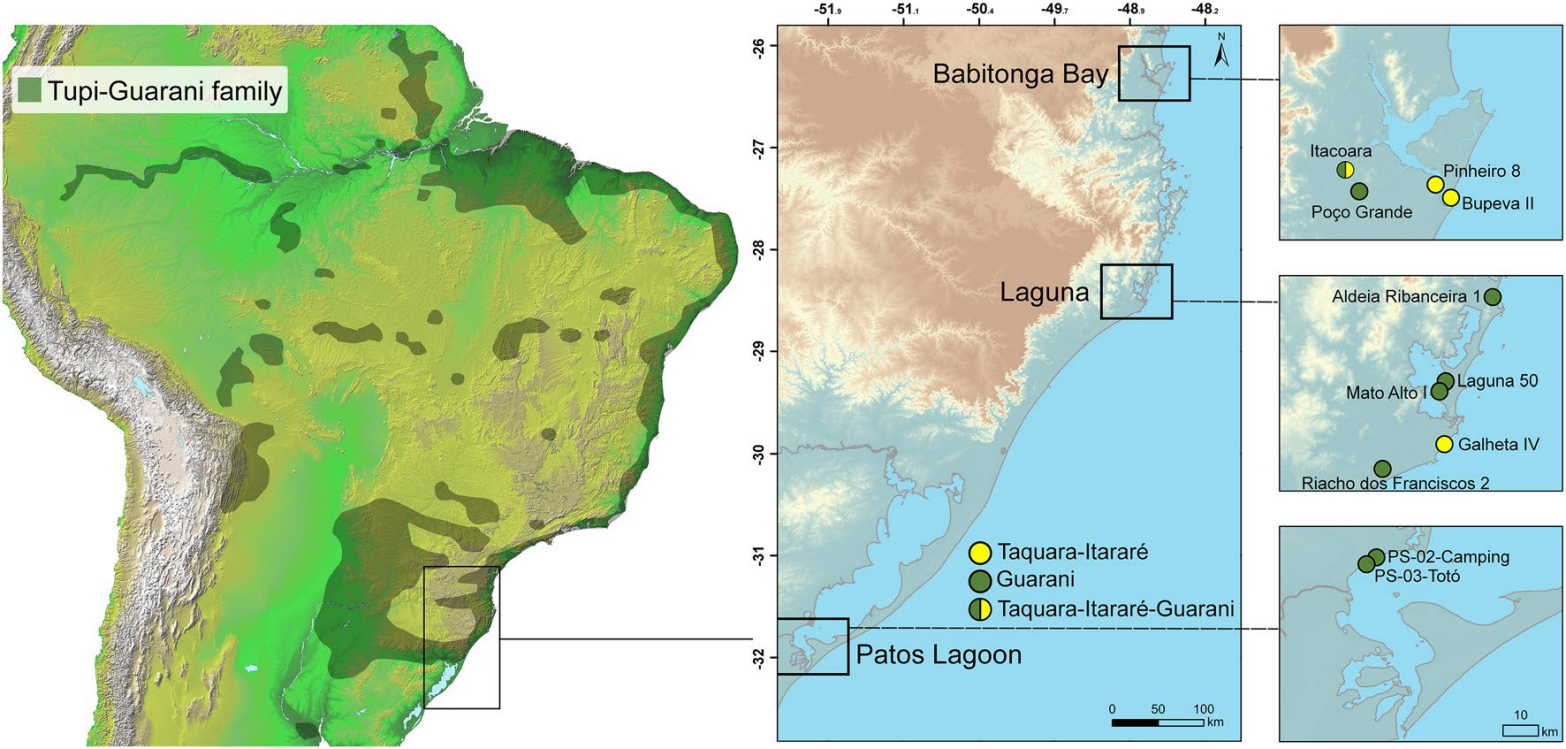
Map of South America, with the distribution of the Tupi-Guarani language family accentuated in dark green and the location of study sites of the Taquara-Itararé (yellow) and Guarani (green) traditions. This map was generated using ArcGIS 10.8, Inkscape and Adobe Illustrator CS6 (see “Methods” for more details).

From ca. 1000 cal BP, new groups associated with the Tupi-Guarani linguistic family began to reach the southern Brazilian coast^9,10^, marking an important cultural change in this region prior to European colonisation in the sixteenth century (Fig. 1). It is well known that the Guarani practiced maize cultivation, which is thought to have helped fuel their demographic expansion from the Northwest Amazon basin over vast areas, as part of one of South America’s largest cultural diasporas^11^. However, due to poor organic preservation, the lack of systematic recovery techniques (e.g. floatation), and a scarcity of human, animal and plant remains at Guarani sites along the Atlantic coast, it is not known whether fishing continued at similar intensity over this period or whether the coastlines and lagoons were given over to crop production. Early chronicles and colonial reports provide valuable, albeit anecdotal, accounts of Guarani subsistence. For example, the German seafarer Hans Staden, held captive by the coastal Guarani in Southern Brazil, described the exploitation of plants and terrestrial prey species (e.g., brocket deer *(Mazama pita)*, tapir *(Tapirus terrestris),* armadillo *(Tolypeutes tricinctus)*, capybara *(Hydrochoerus hydrochaeris)*, etc.) and fishing techniques using nets, bows and arrows^12^.

Historical accounts documented maize cultivation on the Atlantic Forest coast of Brazil at the time of first European arrival^12–14^, mainly in relation to the preparation of *Cauim*: a fermented alcoholic beverage^15,16^. However, it is unclear to what extent maize was used in pre-colonial times. Modern genetic and linguistic evidence suggests a strong connection between the dispersal of maize and the expansion of pre-colonial groups including the Macro-Jê and Tupi-Guarani language families, most recently at ca. 700 cal BP^17,18^. The Guarani expansion territory (Fig. 1) along the South Brazilian coast seems especially connected to the spread of popcorn maize, which has high diversity in Santa Catarina^17,19^. Nonetheless, direct evidence for the antiquity and consumption of maize in pre-colonial Southeastern Brazil is severely limited to occasional finds of microfossils (i.e. phytoliths, starch and pollen) from both the inland^20–23^, and the coast^3,24,25^, an approach not without methodological limitations^26^. Stable isotope analysis of human remains from inland Guarani has highlighted the dietary importance of maize^20,27,28^ but in coastal settings this approach is limited by the near absence of skeletal remains. The few isotope studies of coastal Guarani in Southern Brazil show some ^13^C-enrichment in collagen^27^ but cannot be securely interpreted as maize consumption, as marine resources would likely produce similar values.

Analysis of residues to reconstruct pottery contents provides a proxy for documenting changes in subsistence practices in coastal Brazil, albeit one constrained by cultural ‘culinary’ practices. Pottery is an abundant find at both Taquara-Itararé and Guarani sites and likely played a major role in everyday domestic activities^15^. Diagnostic potsherds can be easily ascribed to cultural traditions allowing a comparative approach to reconstruct their use through time. To investigate, we extracted lipids from 154 Guarani and 55 Taquara-Itararé pottery sherds from 11 sites in Southern Brazil (Table 1, Supplementary Information 1: Table S1). Samples were subjected to gas chromatography-mass spectrometry (GC–MS) and GC-combustion-isotope ratio-MS (GC-C-IRMS). These data were combined with those previously reported from the Galheta IV site (Taquara-Itararé; 15 sherds)^7^ and the Laguna 50 site (Guarani; 20 sherds)^29^. Clear typological differences between Taquara-Itararé and Guarani pottery provided the basis of our sample selection and charcoal directly associated with the potsherds at several sites was radiocarbon dated to confirm the chronological robustness (Supplementary Information 1: Table S2). While the fragmented nature of the sherds did not allow reconstruction of vessel shapes, discrimination based upon provenance and characteristics such as colour, thickness, temper and rim type helped avoid duplicate sampling of the same vessel. We hypothesise that continued intensive marine exploitation should be evident through the presence of aquatic-derived compounds in these vessels. Such compounds have been previously identified on Taquara-Itararé pottery from this region^7,30–32^ but Guarani pottery has never been investigated using this approach.

**Table 1:** A summary of sites, sherds, aquatic biomarkers and Δ^13^C_18:0–16:0_ values.

Period and site	Site description	Radiocarbon date	Calendar date (cal BP)	Sherds	> 5 ug g^−1^ lipid, *n*	Aquatic biomarkers (%)	Δ^13^C_18:0–16:0_ mean
Taquara-Itararé							
Bupeva II	Coastal site on Ilha de São Francisco do Sul	–		34	34	18	− 0.5
Pinheiro 8	Estuary site on the Pinheiros river, Babitonga Bay	–		5	5	–	− 1.0
Galheta IV	Coastal site near Laguna	950 ± 40 (Beta-280012) 980 ± 40 (Beta-211734) 1070 ± 40 (Beta-280011) 1360 ± 40 (Beta-280010)	1150–900	11	11	36	− 1.7
Itacoara	15 km inland from Babitonga bay, on the Piraí river	1250 ± 30	900	16	16	13	− 1.6
Guaraní							
Itacoara	15 km inland from Babitonga bay, on the Piraí river	–		8	8	25	− 1.7
Poço Grande	16 km inland from Babitonga Bay, on the Piraí river	–		21	21	–	− 4.6
Laguna 50 (SC-LGN-50)	Estuary site on the shore of Imaruí lagoon, Laguna	–		45	44	7	− 4.5
Mato Alto I	Estuary site on the shore of Imaruí lagoon, Laguna	–		20	15	20	− 1.7
Aldeia Ribanceira I	Coastal site at Imbituba	502 ± 40 (LT22562) 404 ± 40 (LT22563) 802 ± 40 (LT22564)	549–341 498–322 762–571	16	12	–	− 3.4
Riacho dos Franciscos II	Estuary site near Garopaba do Sul, on the shore of the Jaguaruna Lagoon	770 ± 40 (LT22559) 461 ± 40 (LT22560)	730–565 534–327	20	20	5	− 4.0
PS-02-Camping	Estuary site on the shore of Patos Lagoon	330 ± 50 (Beta-234205)	491–155	6	6	17	− 1.5
PS-03-Totó	Estuary site on the shore of Patos Lagoon	530 ± 40 (Beta-237665) 510 ± 40 (Beta-282128)	621–490 553–461	18	18	6	− 2.1

The Taquara-Itararé sites studied here are located in the State of Santa Catarina and include coastal and inland locations in the vicinity of large estuary systems (Fig. 1). Bupeva II is a Sambaqui with a Taquara-Itararé ceramic component^33^. Galheta IV (ca. 1150–900 cal BP) is a funerary site^7^. Both sites are situated directly on the coast and presented abundant evidence for marine and estuary subsistence focus through zooarchaeological assemblages, bone isotope analysis and organic residue analysis^7,33–35^. Pinheiro 8 is also a Sambaqui, located close to the coast (3 km) along an estuary channel^36,37^. Itacoara (ca. 900 cal BP) is a multicultural site with a pre-ceramic phase and both Taquara-Itararé and Guarani pottery in the more recent layer. The site is situated along the Piraí river, some 25 km from the coast. Zooarchaeological evidence and the presence of fishhooks in the ceramic levels of the site attest to the importance of fishing at this locality^33, 38^. Most Guarani sites are located on the shores of estuary systems (Babitonga Bay, Laguna and Patos Lagoon), rather than directly on the coast. Poço Grande is situated along the Piraí river, approximately 22 km from the coast and is the only Guarani site in the vicinity of Babitonga Bay^33^. At the sites of Laguna 50^39^, Mato Alto 1^40,41^ and Riacho dos Franciscos 2^42–44^ organic remains were rare. A bone hook, potentially used for fishing, was found at the coastal site of Aldeia Ribanceira 1^45^. Further to the south, fishing was an important activity at the PS-02-Camping (ca. 491–155 cal BP) and PS-03-Totó sites (ca. 621–461 cal BP) on the eastern shore of Patos Lagoon in the State of Rio Grande do Sul. Estuary fish are abundant at these sites, especially whitemouth croaker *(Micropogonias furnieri)* and black drum *(Pogonias cromis)*. Botanical remains are absent^46–49^.

## Results and discussion

Overall, lipids clearly derived from aquatic sources, such as marine and freshwater fish, were more abundant in the Taquara-Itararé pottery compared to Guarani (Table 1, Supplementary Dataset S1). In total, 17% of the Taquara-Itararé pottery samples either yielded long-chain C_18_ and C_20_ ω-(o-alkylphenyl) alkanoic acids (APAAs) or dihydroxy acids (DHAs) formed from mono-, di- and tri-unsaturated fatty acids present in aquatic oils^30,32,50^, compared to only 7% of the Guarani pottery samples. This difference is corroborated by the presence of isoprenoid fatty acids (TMTD, pristanic and phytanic acid) which were significantly more abundant in Taquara-Itararé than in Guarani pottery (Mann Whitney U = 1947, *p* = < 0.05), and by the ratio of phytanic acid stereoisomers (%SRR), also significantly higher in the Taquara-Itararé vessels (Mann Whitney U = 3243, *p* = < 0.05; Supplementary Dataset S1, both indicative, although not diagnostic, of aquatic species^51^. Interestingly, six samples from two Taquara-Itararé coastal sites yielded aquatic derived APAAs but have %SRR values (20 to 47%) and TMTD/phytanic acid ratios (0.34–1.63) more comparable to shellfish than either freshwater or marine fish and mammals (Supplementary Dataset S2). Despite a difference between cultural phases, the overall frequency of aquatic lipid biomarkers was much lower than in other studies of pottery use by coastal economies (e.g.^52^), even in Taquara-Itararé samples where high frequencies of aquatic biomarkers were previously reported^30–32^. Although this finding seems unexpected given the extreme marine isotopic signature in human bone collagen from coastal Taquara-Itararé, Bayesian isotope mixing models applied to the bone isotope data show that substantial amounts of terrestrial animals and plants to total diet can be accommodated^4^. Factors affecting the formation and preservation of aquatic biomarkers in pottery may also be relevant.

**Figure 2:**
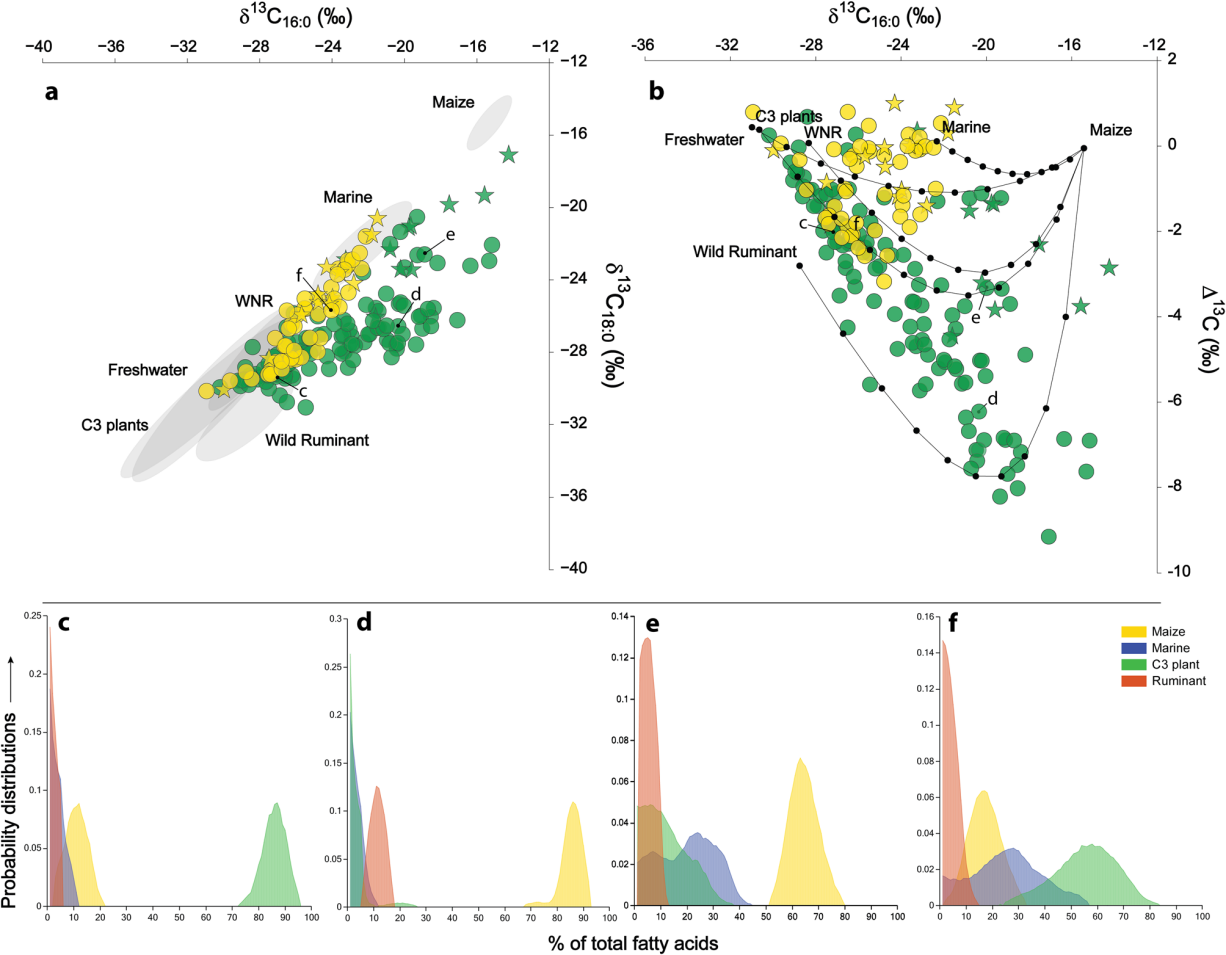
(**a**) compound specific isotope values of fatty acids C_16:0_ and C_18:0_ against modern reference values expressed as 68% confidence ellipses (Supplementary Dataset S2), and (**b**) Δ^13^C_18:0–16:0_ from Taquara-Itararé pottery (yellow) and Guarani pottery (green), stars reflect the presence of aquatic biomarkers. (**c**–**f**) Probability distributions of Bayesian modeling of Guarani samples (**c**) SCLGN050-214, (**d**) SCLGN050-186 and (**e**) SCLGN050-177, and Taquara-Itararé sample (**f**) BP-9768 shown as % of total fatty acids (Supplementary Table S3).

To further establish changes in vessel use, we compared the carbon isotopic values (δ^13^C) of palmitic (C_16:0_) and stearic acid (C_18:0_) in each of the Taquara-Itararé and Guarani lipid samples. These data provide a more quantitative approach to the reconstruction of pottery use and assessing inputs. Plots of the δ^13^C_16:0_ against δ^13^C_18:0_ and consideration of the offset between these values (Δ^13^C_18:0–16:0_) shows clear differences between Taquara-Itararé and Guarani potsherds (Fig. 2a,b). As expected, many of the Taquara-Itararé samples plot within the ranges of authentic marine, estuarine and freshwater fish oils, corresponding generally to the geographic location of sites, but isotope values indicative of ruminant fats and C_3_ plant oils, or mixtures of these, were also obtained. In contrast, the Guarani isotope data shows an unusual distribution with large Δ^13^C_18:0–16:0_ offsets. Such values are usually attributed to ruminant dairy fats^53^, but dairying was not practised in Southeastern Brazil prior to European colonisation and the introduction of domesticated ruminants. Instead, we propose that the Guarani pottery samples with relatively enriched δ^13^C_16:0_ values are instead the result of the processing of maize (a C_4_ plant) in these vessels. Maize kernel oil has very low relative concentrations of the C_18:0_ acid compared to other products (Supplementary Information 1: Fig. S1a,b), so progressive mixtures of maize with even small quantities of C_3_ animal fats are predicted to produce decreasing Δ^13^C_18:0–16:0_ values (Fig. 2b). Such a pattern is not predicted if C_4_-fed animals were a source of the lipids. Some of the highest palmitic/stearic acid ratios (P/S, up to 10.7) occur in samples with large Δ^13^C_18:0–16:0_ (Pearson r = − 0.48, df = 128, *p* ≤ 0.05), further supporting the plant origin of these samples.

To further quantify the C_4_ plant input, we also considered the δ^13^C value of oleic acid (C_18:1_), where it was measurable, as this component is relatively abundant in maize oil (Supplementary Information 1: Fig. S1) compared to animal products. The results (Supplementary Dataset S1) confirm a corresponding increase in δ^13^C_16:0_ and δ^13^C_18:1_ values in the Guarani samples supporting our interpretation (Pearson r = 0.76, df = 18, *p* ≤ 0.05). Using a concentration dependent Bayesian mixing model^54^ (see also Supplementary FRUITS Files 1–2), we were able to provide probabilistic estimates of maize versus marine and C_3_ terrestrial contributions for selected samples using all three fatty acid isotope proxies. This analysis supports the interpretation that Guarani samples with large Δ^13^C_18:0–16:0_ offsets derived the majority of their fatty acids from maize (Fig. 2d,e) compared to other products (Fig. 2c,f). This estimation translates to an even greater contribution by weight if the relative concentration of fatty acids in dry tissues are considered. Using this approach, it is evident that C_4_ plant processing was practised at all Guarani sites regardless of their location. Interestingly, this pattern was less extreme at the Itacoara (Babitonga Bay) and at the Patos Lagoon sites (PS-03-Totó and PS-02-Camping), which may be related to site function (e.g. fishing/hunting camps versus residential village sites). Instead C_3_ products, including plants and ruminant fats, were more prominent in those sites, pointing to some regional diversity. Maize was most frequently present in Guarani pots from the Laguna region (i.e., at Laguna 50, Riacho dos Franciscos 2, and Aldeia Ribanceira 1; see Table 1, Supplementary Dataset S1) as well as at Poço Grande near Babitonga Bay.

Previously it has been proposed that the long-chain C_32_ *n-*alkanol (*n*-dotriacontanol) serves as a useful lipid biomarker for maize in archaeological pottery as this compound is rare in other native, edible American plants^55^. While long-chain alkanols were identified in solvent extracts of 15 Guarani samples, most samples only yielded trace amounts of *n*-dotriacontanol. Exceptions are samples RDF2-641 and RDF2-831 where abundant *n*-alkanols with 28–36 carbon atoms were observed, providing further corroboration of maize in these samples (Supplementary Dataset S1). Nonetheless, this marker was absent in most of the samples identified as maize-rich based on the isotopic criteria outlined above, limiting the applicability of the approach, at least in these contexts. Other non-specific plant biomarkers (sterols and terpenes) were observed in both Guarani and Taquara-Itararé samples as well as distributions of C_18_ APAAs, consistent with leafy plants^50^ (Supplementary Dataset S1). High abundances of lauric acid (C_12:0_) in several samples suggests the processing of palm oil^56^ (Supplementary Dataset S1, Fig. S1c). The processing of starchy plants such as manioc would be expected to be almost analytically invisible using this approach, due to their trace lipid content (< 0.3% by dry weight,^57^) but may well have contributed. Levoglucosan, a starch or cellulose pyrolysis product previously identified in archaeological vessels^58^ was absent in all solvent extracted samples. Scanning electron microscopy (SEM) analysis was applied to a limited number (n = 6) of carbonised crusts on the pottery surfaces but yielded no interpretable results.

Bacteriohopanes (C_30–33_), previously interpreted as fermentation markers^59^, are present in most Guarani samples (Supplementary Dataset S1), although this interpretation is inconclusive without corroborating evidence^60^. The production of the fermented beverage *Cauim* with maize, manioc and other plants, is documented in historic accounts of the Guarani^16^, especially in large conical jars (i.e., *Cambuchí)* that were partially dug into the ground. However, due to the fragmented nature of the pottery assemblage it is not possible to reconcile the shape of vessels with their function^15^. Nonetheless, differences between plain (n = 88) and decorated (n = 70) Guarani sherds (Mann Whitney U = 18, *p* = 0.056), with the former showing more enriched δ^13^C_18:1_ values, may hint at correlations between form and function. There were no differences, however, in the frequency of bacteriohopanes between decorated and undecorated sherds (*t*(0.26), *p* = 1.975).

## Conclusions

Previous archaeological investigations have shown that the maritime economy and diet of people living along the Atlantic coast of Southern Brazil (∼ 6700 to ∼ 1000 cal BP) was remarkably resilient, despite a series of demographic, cultural and environmental changes^4^. People bringing new ideas, artefacts and foodstuffs to the coast appear to have been rapidly absorbed into an economy principally focused on coastal and estuarine exploitation of molluscs, fish and mammals, including deep ocean fishing. Both high biological productivity of the Atlantic coastal ecotone and deep-rooted local ecological knowledge are likely contributing factors to long-term continuity of maritime economies in this region. These historical data therefore underpin a compelling argument to inform future coastal management strategies and advocate the protection of artisanal fisheries. Nevertheless, based on the use of pottery, we show that during the phase that Guarani groups occupied the region, maize became a significant commodity. Indeed, while both maize^20^ and pottery^61^ were present in southern South America for several thousand years, maize cultivation did not intensify until the arrival of the Guarani in the region. Among the Guarani it was widely used, consumed as a fermented beverage, and likely mixed with other foodstuffs as part of routine culinary practices. This finding is supported by the sparse number of isotopic analyses, so far, conducted on coastal Tupi-Guarani human remains^27,62^ and zooarchaeological studies^63^, that also show a departure from previous coastal oriented economies and, at least, a broadening of the subsistence base at this juncture.

Our findings stress the importance of maize to fuel the cultural expansion of the Guarani, and it may be that the crop, along with other economic practices, was of such inherent cultural significance to the Guarani that abandoning it in favour of fishing was inconceivable. We know, for example, that political alliances and social status were closely related to feasting and the preparation of *Cauim*^11,12^. In turn, the Guarani diaspora was of importance to the genetic and geographic variation of maize in South America^17^. Indeed, the longer-term consequences of the Guarani expansion, which eventually encompassed the entire coast of Brazil, are potentially profound. The broadening of subsistence practices and widespread cultivation of maize may have alleviated some anthropogenic pressure on marine and estuarine systems that had previously been intensely exploited for millennia, and as a result may have led to a loss of maritime indigenous knowledge. Our study also highlights the problem of ‘shifting baselines’ when using archaeological and historical data to inform modern policies, and the danger of assuming that ‘prehistory’ was culturally and economically static, as it is frequently presented in colonial discourse. Rather, knowledge of the long-term dynamics of regional socio-ecological systems, their specific limits and environmental responses, should be useful for developing the routes and interventions towards future desirable scenarios.

## Methods

### Sampling

For this research a total of 224 samples were analysed, originating from 11 archaeological sites (3 of which containing Taquara-Itararé pottery, and 8 of which containing Guarani pottery) see Supplementary Information 1: Table S1. Sherds were assigned to cultural traditions based on AMS radiocarbon (^14^C) dating of associated organic material or based on relative chronology and pottery typology. Permits for organic residue analysis and radiocarbon dating were obtained from the Instituto do Patrimônio Histórico e Artístico Nacional (IPHAN, protocol 01510.000608/2021-80, 01512.000594/2020-01, 01510.000612/2020-67, 01510.000422/2022-10). To compare, modern references of plants and animals (bone) were obtained from Brazil and Argentina (Supplementary Information 1: Table S2). Plant samples from Brazil were registered in the Sistema Nacional de Gestão do Patrimônio Genético e do Conhecimento Tradicional Associado (SisGen, shipping no. RF54A7C) according to Brazilian law no. 13.123, of 20 May 2015 and its regulations.

### Organic residue analysis

A total of 224 ceramic sherds from the Brazilian Atlantic Forest coast were sampled for organic residue analysis. Ceramic powder samples (ca. 1 g) were collected by drilling into the pottery fabric (with a Dremel handheld drill) after removing a small layer (~ 1 mm) to avoid contamination from handling and from the burial environment. All samples were extracted using acidified methanol following established protocols^64^. In short, methanol (4 mL) was added to the homogenised ceramic samples (1 g). After sonication (15 min), 800 μL of sulfuric acid was added to the samples after which they were placed in a heating block for 4 h at 70 °C. Lipids were then extracted using n-hexane (3 × 2 mL). Modern references from Argentina were subjected to experiments in order to obtain APAA’s according to established protocols^50^ and were heated at 270 °C for 5 h before transport and extraction in York. These experimental samples, as well as modern reference plants from Brazil, were then extracted following the above procedure. Bone samples, also used as references, were crushed to a homogenised powder (1 g) and solvent washed (3 × 2 mL dichloromethane/methanol 2:1 v/v wash) before extraction following the above-described steps. A selection of 107 ceramic samples were also subjected to solvent extraction following established procedures^65^. Briefly, 1 g of ceramic powder was extracted using a mixture of dichloromethane/methanol 2:1 v/v wash (3 × 2 mL). All acid and solvent extracted samples were also derivatized using BSTFA. All samples were analysed by GC-flame ionisation detection (GC-FID), GC-MS, and GC-C-IRMS (see Supplementary Information 1).

### Statistical analysis and Bayesian modelling

Statistical analysis was performed using R studio (version 2022.07.2) and Past (version 4.03). Compound specific isotope data of ceramic samples was modelled using the Bayesian mixing model FRUITS (^54^, Beta version 3.0: http://sourceforge.net/projects/fruits/; models attached as Supplementary FRUITS File 1–2). Palmitic (δ^13^C_16:0_), stearic (δ^13^C_18:0_) and oleic acid (δ^13^C_18:1_) were used as proxies. Four food groups (maize, marine, C_3_ plants and ruminants) were selected as potential sources (Supplementary Information 1: Table S3) and carbon isotope values were obtained from authentic reference fats of modern and historic animals and plants both from the literature and obtained for this study (Supplementary Dataset S2). Uncertainties of the isotope values were incorporated into the model through non-conservative covariance matrices (Supplementary FRUITS File 1 and 2). Concentrations of palmitic, stearic and oleic acid were obtained from the USDA Food Composition Database^57^, and from authentic reference fats (Supplementary Dataset S2) and are expressed as % of total fatty acids.

### Mapping

The map in Fig. 1 was generated using ArcGIS 10.8 (https://desktop.arcgis.com/en/), Inkscape (https://inkscape.org/) and Adobe Illustrator CS6 (https://www.adobe.com/es/products/illustrator.html) on data publicly available from Natural Earth (https://www.naturalearthdata.com/), National Institute for Space Research—INPE (http://terrabrasilis.dpi.inpe.br/), CGIAR Consortium for Spatial Information (https://cgiarcsi.community/data/srtm-90m-digital-elevation-database-v4-1/) and NASA/JPL-Caltech (adapted from https://www.jpl.nasa.gov/images/pia03388-south-america-shaded-relief-and-colored-height).

### Supplementary Information

Supplementary Information 1.

Dataset S1.

Dataset S2.

Supplementary Information 2.

Supplementary Information 3.

## Data Availability

All data presented in this article are made available in the supporting information.

## References

[1] Strassburg BBN (2020). Global priority areas for ecosystem restoration. Nature.

[2] Begossi, A. Cultural and ecological resilience among Caiçaras of the Atlantic Forest coast and caboclos of the Amazon. *Linking social and ecological systems for resilience and sustainability, *129–157 (The Beijer International Institute of Ecological Economics, 1998).

[3] Scheel-Ybert R, Boyadjian C (2020). Gardens on the coast: Considerations on food production by Brazilian shellmound builders. J. Anthropol. Archaeol..

[4] Toso A (2021). Fishing intensification as response to Late Holocene socio-ecological instability in southeastern south America. Sci. Rep..

[5] Iriarte J, DeBlasis P, De Souza JG, Corteletti R (2017). Emergent complexity, changing landscapes, and spheres of interaction in southeastern south America during the middle and late holocene. J. Archaeol. Res..

[6] Corteletti R, Dickau R, DeBlasis P, Iriarte J (2015). Revisiting the economy and mobility of southern proto-Jê (Taquara-Itararé) groups in the southern Brazilian highlands: Starch grain and phytoliths analyses from the Bonin site, Urubici, Brazil. J. Archaeol. Sci..

[7] Colonese AC (2014). Long-term resilience of late Holocene coastal subsistence system in southeastern South America. PLoS ONE.

[8] Gilson S-P, Lessa A (2021). Capture, processing and utilization of sharks in archaeological context: Its importance among fisher-hunter-gatherers from southern Brazil. J. Archaeol. Sci. Rep..

[9] Bonomo M, Costa Angrizani R, Apolinaire E, Noelli FS (2015). A model for the Guarani expansion in the La Plata Basin and littoral zone of southern Brazil. Quat. Int..

[10] Milheira RG, Wagner GP (2014). Arqueologia Guarani no Litoral Sul do Brasil.

[11] Iriarte J (2017). Out of amazonia: Late-holocene climate change and the tupi-guarani trans-continental expansion. Holocene.

[12] Staden H (2008). Hans Staden’s True History: An Account of Cannibal Captivity in Brazil.

[13] de Léry J (1972). Histoire d’'un Voyage Fait en la Terre de Brésil.

[14] A. Thevet, *Les Français en Amérique pendant la deuxième moitié du XVI siècle: Le Brésil et les Brésiliens, par André Thevet* (Presses Universitaires de France, 1953).

[15] Brochado JP (1991). What did the Tupinambá cook in their vessels? An humble contribution to ethnographic analogy. Rev. Arqueol..

[16] Noelli FS, Brochado JP (1998). The cauim and the beverages among the guarani and the tupinambá: Equipments, preparation techniques and consumption. Rev. Museu Arqueol. Etnol..

[17] Costa FM (2022). Maize dispersal patterns associated with different types of endosperm and migration of indigenous groups in lowland South America. Ann. Bot..

[18] Kistler L (2018). Multiproxy evidence highlights a complex evolutionary legacy of maize in South America. Science.

[19] Costa FM, de Silva NCA, Ogliari JB (2017). Maize diversity in southern Brazil: Indication of a microcenter of *Zea mays* L.. Genet. Resour. Crop Evol..

[20] Iriarte J (2004). Evidence for cultivar adoption and emerging complexity during the mid-Holocene in the La Plata basin. Nature.

[21] Iriarte J, Christopher Gillam J, Marozzi O (2008). Monumental burials and memorial feasting: An example from the southern Brazilian highlands. Antiquity.

[22] Behling H, Pillar VD, Bauermann SG (2005). Late quaternary grassland (Campos), gallery forest, fire and climate dynamics, studied by pollen, charcoal and multivariate analysis of the São Francisco de Assis core in western Rio Grande do Sul (southern Brazil). Rev. Palaeobot. Palynol..

[23] Schneider, F., Corteletti, R., Machado, N. T. G., Stülp, S. Arqueobotânica guarani: A presença de grãos de amido, fitólitos e endocarpos carbonizados no sítio RS-T-114, Bacia do Rio Forqueta, Rio Grande do Sul, Brasil, in *XIX Congresso de Arqueologia Argentina*, 1001–1006 (Universidad Nacional de Tucumán, 2016).

[24] Boyadjian C, Eggers S, Scheel-Ybert R, Hardy K, Kubiak-Martens L (2016). Evidence of plant foods obtained from the dental calculus of individuals from a Brazilian shell mound. Wild Harvest: Plants in the Hominine and Pre-Agrarian Human Worlds.

[25] Wesolowski V, de Souza SMFM, Reinhard KJ, Ceccantini G (2010). Evaluating microfossil content of dental calculus from Brazilian Sambaquis. J. Archaeol. Sci..

[26] Mercader J (2018). Exaggerated expectations in ancient starch research and the need for new taphonomic and authenticity criteria. Facets (Ott).

[27] Loponte D, Carbonera M (2021). From the Atlantic coast to the lowland forests: Stable isotope analysis of the diet of forager–horticulturists in southern Brazil. Int. J. Osteoarchaeol..

[28] Bonomo M, Aceituno FJ, Politis GG, Pochettino ML (2011). Pre-Hispanic horticulture in the Paraná Delta (Argentina): Archaeological and historical evidence. World Archaeol..

[29] Chervenka K (2019). Organic Residue Analysis Reveals Possible Dietary Reformation Connected to the Tupi Guarani Dispersal to the Southern Coast of Brazil.

[30] Hansel FA, Copley MS, Madureira LAS, Evershed RP (2004). Thermally produced ω-(o-alkylphenyl)alkanoic acids provide evidence for the processing of marine products in archaeological pottery vessels. Tetrahedron Lett.

[31] Hansel FA, Evershed RP (2009). Formation of dihydroxy acids from Z-monounsaturated alkenoic acids and their use as biomarkers for the processing of marine commodities in archaeological pottery vessels. Tetrahedron Lett..

[32] Hansel FA, Bull ID, Evershed RP (2011). Gas chromatographic mass spectrometric detection of dihydroxy fatty acids preserved in the “bound” phase of organic residues of archaeological pottery vessels. Rapid Commun. Mass Spectrom..

[33] Bandeira DR (2004). Ceramistas Pre-coloniais da Baia da Babitonga, SC: Arqueologia e Etnicidade.

[34] DeBlasis, P., Farias, D. S., Kneip, A. Velhas tradições e gente nova no pedaço: Perspectivas longevas de arquitetura funerária na paisagem do litoral sul catarinense. *Rev. Mus. Arqueol. Etnol.* 109 (2014).

[35] Fossile T (2019). Pre-Columbian fisheries catch reconstruction for a subtropical estuary in South America. Fish Fish..

[36] Piazza W, Prous A (1977). Documents Pour la Préhistoire du Brésil Méridional: 2: l’état de Santa Catarina.

[37] Tiburtius, G., Bigarella, J. J. & Bigarella, I. K*. Contribuição ao Estudo dos Sambaquis do Litoral de Santa Catarina. II – Sambaqui do Rio Pinheiros n.o 8*. vol. IX, 141–197 (Impressora Paranaense, 1954).

[38] Tiburtius, G., Bigarella, I. K. & Bigarella, J. J. Nota prévia sobre a jazida paleoetnográfica de itacoara (Joinville, Estado de Santa Catarina), in *Arquivos de Biologia e Tecnologia*, vol. V–VI (Impressora Paranaense, 1951).

[39] Farias, D. E. Programa de salvamento arqueológico pré-histórico e educação patrimonial na área de duplicação da BR -101 trecho Ponte de Cabeçudas, Laguna – SC. Projeto de Pesquisa (2012).

[40] Rohr, J. A. *Sítios Arqueológicos do Município Sul-Catarinense de Jaguaruna*, vol. 22 (Instituto anchietano de pesquisas, 1969).

[41] Giannini PCF (2010). Interactions between sedimentary evolution and prehistoric human occupation in the south-central coast of Santa Catarina, Brazil. Bol. Mus. Para. Emílio Goeldi. Ciênc. Hum..

[42] Farias, D. E., Demathe, A., Alves, L. & Machado, G. B. *Programa de Resgate Arqueológico do Sítio Riacho dos Franciscos II, Município de Jaguaruna – SC* (2021).

[43] Milheira, R. G. & Deblasis, P. O território Guarani no litoral sul catarinense: Ocupação e abandono no limiar do período colonial. *Rev. Arqueol. Am.* 147–182 (2013).

[44] Milheira, R. G. *Teritório em conflito: Arqueologia Guarani no litoral sul-catarinense*. (Editoria Prismas, 2018).

[45] Demathe, A., Oliveira, A. R., Alves, L. & Machado, G. B. Programa de Gestão do Patrimônio Arqueológico na Área de Implantação do Loteamento Mirante da Baleia – salvamento do sítio arqueológico Aldeia Ribanceira I, Município de Imbituba-SC (2023).

[46] Milheira RG (2008). Um modelo de ocupação regional Guarani no sul do Brasil. Rev. Mus. Arqueol. Etnol..

[47] Milheira RG (2014). Arqueologia Guarani na Planície Sudoeste da Laguna dos Patos e Serra do Sudeste.

[48] Milheira RG, Dos Santos J (2020). Dos potes ao território: O desafio metodológico brochadiano em dois contextos Guarani. Habitus.

[49] Milheira RG, Ulguim PF (2008). Uma contribuição para a zooarqueologia em sítios Guarani do litoral sul do Brasil, Laguna dos Patos, Pelotas-RS: Estratégias de assentamento, aspectos alimentares e função de sítio. Clio.

[50] Bondetti M (2021). Investigating the formation and diagnostic value of ω-(o-alkylphenyl)alkanoic acids in ancient pottery. Archaeometry.

[51] Lucquin A, Colonese AC, Farrell TFG, Craig OE (2016). Utilising phytanic acid diastereomers for the characterisation of archaeological lipid residues in pottery samples. Tetrahedron Lett..

[52] Admiraal M, Lucquin A, von Tersch M, Craig OE, Jordan PD (2020). The adoption of pottery on Kodiak Island: Insights from organic residue analysis. Quat. Int..

[53] Copley MS (2003). Direct chemical evidence for widespread dairying in prehistoric Britain. Proc. Natl. Acad. Sci. U. S. A..

[54] Fernandes R (2018). Reconstruction of prehistoric pottery use from fatty acid carbon isotope signatures using Bayesian inference. Org. Geochem..

[55] Reber EA, Dudd SN, Van der Merwe NJ, Evershed RP (2004). Direct detection of maize in pottery residues via compound specific stable carbon isotope analysis. Antiquity.

[56] Copley MS (2001). Detection of palm fruit lipids in archaeological pottery from Qasr Ibrim, Egyptian Nubia. Proc. Biol. Sci..

[57] U.S. Department of Agriculture, Agricultural Research Service, Data from “USDA Food and Nutrient Database for Dietary Studies 2011–2012”. *Food Surveys Research Group Home Page* (2014) Available at https://fdc.nal.usda.gov/. Deposited 10 June 2022.

[58] Shoda S (2018). Molecular and isotopic evidence for the processing of starchy plants in Early Neolithic pottery from China. Sci. Rep..

[59] Correa-Ascencio M, Robertson IG, Cabrera-Cortés O, Cabrera-Castro R, Evershed RP (2014). Pulque production from fermented agave sap as a dietary supplement in Prehispanic Mesoamerica. Proc. Natl. Acad. Sci. U. S. A..

[60] Craig OE (2021). Prehistoric fermentation, delayed-return economies, and the adoption of pottery technology. Curr. Anthropol..

[61] Milheira RG, Gianotti Garcia C (2018). The earthen mounds (Cerritos) of southern Brazil and uruguay. Encyclopedia of Global Archaeology.

[62] Colonese AC (2020). Stable isotope evidence for dietary diversification in the pre-Columbian Amazon. Sci. Rep..

[63] Rosa AO (2010). Arqueofauna de um sítio de ocupação pré-histórica Guarani no município de Porto Alegre, Río Grande do Sul. Pesqui. Antropol..

[64] Papakosta V, Smittenberg RH, Gibbs K, Jordan P, Isaksson S (2015). Extraction and derivatization of absorbed lipid residues from very small and very old samples of ceramic potsherds for molecular analysis by gas chromatography-mass spectrometry (GC-MS) and single compound stable carbon isotope analysis by gas chromatography-combustion-isotope ratio mass spectrometry (GC-C-IRMS). Microchem. J..

[65] Evershed RP, Heron C, Goad J (1990). Analysis of organic residues of archaeological origin by high-temperature gas chromatography and gas chromatography mass spectrometry. Analyst.

