# Supplementary material for: Chemical analysis of pottery reveals the transition from a maritime to a plant-based economy in pre-colonial coastal Brazil
Source: Sci Rep. 2023 Oct 5;13:16771. doi: 10.1038/s41598-023-42662-5 (PMC10556129; doi:10.1038/s41598-023-42662-5)
Supplement: Supplementary file 1 — Supplementary Information 1. [file 41598_2023_42662_MOESM1_ESM.docx]

**Supplementary Information 1**

**Chemical analysis of pottery reveals the transition from a maritime to a plant-based economy in pre-colonial coastal Brazil**

Marjolein Admiraal, Andre C. Colonese, Rafael G. Milheira, Dione da Rocha Bandeira, Alexandro Demathe, Adriana M. Pereira dos Santos, Thiago Fossile, Helen M. Talbot, Manon Bondetti, Alexandre Lucquin, Javier Montalvo-Cabrera, Luciano Prates, Alejandro Serna and Oliver E. Craig 

Instrumentation

**Gas Chromatography - Flame Ionization Detector (GC-FID).** Quantification and general screening of the samples was done by GC-FID, using an Agilent 7890A gas chromatograph (Agilent Technologies, Cheadle, Cheshire, UK). The splitless injector was maintained at 300°C and 1 µL of sample diluted in hexane was injected into the GC. A 100% Dimethylpolysiloxane DB-1 (15 m x 320 µm x 0.1 µm; J&W Scientific, Folsom, CA, USA) column was used and the carrier gas was helium and set at a constant pressure of 16.6 psi. The flow rate was constant and set to 2 mL min^-1^. The temperature was set at 100°C for 2 min, increased by 20°C min^-1^ until a temperature of 325°C was reached which was maintained for 3 min. Total run time per sample was 16.25 min.

**GC - Mass Spectrometry (GC-MS).** GC-MS analysis was carried out on several instruments: 1) an Agilent 7890A series GC connected to an Agilent 5975C Inert XL mass-selective detector with a quadrupole mass analyzer (Agilent Technologies, Cheadle, Cheshire, UK); 2) a Trace Ultra 1310 GC, coupled with an ISQ MS (Thermo Fisher, Bremen, Germany); and 3) a Perkin Elmer CLARUS 690 SQ 8T GC-MS using the same GC configuration and temperature program. Reproducibility between instruments and runs was tested by measuring standard lipid mixtures added as a standard to each run.

A DB-5ms (5%-phenyl)-methylpolysiloxane column (30 m × 0.250 µm × 0.25 µm; J&W Scientific, Folsom, CA, USA) was used for scanning. The injector was set to splitless mode and kept at 300°C. And the GC column was inserted directly into the MS ion source. The carrier gas was helium with a flow rate of 3 mL/min. Spectra were obtained by scanning between *m/z* 50 and 800 and the MS ionization energy was 70 eV. The temperature was set to 50°C for 2 min and then raised by 10°C min^-1^. Finally, the temperature was held for 15 min at 325°C. To better quantify aquatic biomarkers (isoprenoid acids and ω-(o-alkylphenyl) alkanoic acids) (1) all acidified-methanol extracted samples extracted were also analyzed using instrument 1 equipped with a DB-23 (50%-cyanopropyl)-methylpolysiloxane column (PN 122-2362; 60 m × 0.250 µm × 0.25 µm; J&W Scientific) and running in SIM mode (single ion monitoring). The injector was set to splitless mode and kept at 300°C. The temperature program was set to 50°C (2 min) and was then raised by 10°C min^-1^ until 100°C, then it was raised by 4°C min^-1^ until 140°C, then by 0.5°C min^-1^ to until 160°C, and finally by 20°C min^-1^ to reach 250°C, there it was kept constant for 10 min. Spectra for the several compounds were obtained by scanning specific ions: *m/z* 74, 87, 213, 270 for 4,8,12-trimethyltridecanoic acid (TMTD); *m/z* 74, 88, 101, 312 for pristanic acid; and *m/z* 74, 101, 171, 326 for phytanic acid. Finally, spectra of ω-(o-alkylphenyl) alkanoic acids (APAA) of carbon length C^16^ to C^22^ were obtained by scanning the ions *m/z* 74, 105, 262, 290, 318, 346. The carrier gas was Helium with a (constant) flow rate of 1.5 mL/min. The quantification of compounds was acquired using MassHunter software (version B.07.01, Agilent Technologies) by the integration of ion *m/z* 87 (saturated and branched fatty acids and TMTD), *m/z* 88 (pristanic acid), *m/z* 101 (phytanic acid), *m/z* 290 (APAA C^18:0^), *m/z* 318 (APAA C^20:0^), and *m/z* 75 (*n-*alkanols).

**GC-combustion-Isotope Ratio MS (GC-c-IRMS).** To obtain the stable isotope values of palmitic (C_16:0_), stearic (C_18:0_) and oleic acid (C_18:1_), we analyzed 194 samples by GC-c-IRMS following existing protocol (2). The instruments used were 1) a Isoprime 100 (Isoprime, Cheadle, UK) linked to an Agilent 7890B Series GC (Agilent Technologies) with an Isoprime GC5 interface (Isoprime), and 2) a Delta V Advantage isotope ratio MS (Thermo Fisher) coupled with a Trace Ultra 1310 GC (Thermo Fisher) and with a GC Isolink II interface (CuO combustion reactor held at 850 °C). The carrier gas was ultra-purity grade Helium and was set at a constant flow rate of 2 mL/min. Parallel acquisition of the molecular data was achieved with instrument 2 by splitting the flow to direct a small part of the sample to an ISQ MS (Thermo Fisher). One µL of sample suspended in hexane was injected at 300°C into a DB-5MS ultra-inert fused-silica column (PN 122-5562UI; 60 m x 250 µm x 0.25 µm; J&W Scientific). The temperature program was set at 50°C (0.5 min), subsequently raised by 25°C min^-1^ to reach 175°C, and finally increased by 8°C min^-1^ to 325°C where it was held for 20 min. Clear resolution and baseline separation of the peaks was achieved.

Eluted products were ionized by electron impact in the MS and ion intensities (*m/z* 44, 45, 46) were recorded for automated computing of the ^13^C/^12^C ratio of each peak in the lipid extracts. Data analysis was carried out with Isodat (version 3.0; Thermo Fisher), IonVantage and IonOS softwares (Isoprime, Cheadle, UK). Results were expressed in per mill (‰) relative to an international standard (V-PDB). The accuracy and precision of the instrument was determined based on expected versus measured δ^13^C values of n-alkanes and n-alkanoic acid ester international standards (Indiana A7 and F8-3). Mean ± S.D. (standard deviation) values were 29.85 ± 0.14‰ for the methyl ester of C_16:0_ (reported mean value vs. V-PDB -29.90 ± 0.03‰), and 23.23 ± 0.14‰ for the methyl ester of C_18:0_ and C_18:1_ (reported mean value vs. VPDB -23.24 ± 0.01‰). All samples were measured in replicate (mean of S.D. 0.13‰ for C_16:0_, C_18:0_, and for C_18:1_). Values were corrected after analysis using a mass balance formula to account for the methylation of the carboxyl group that occurs during acidified-methanol extraction. Further corrections were based on a comparison with standard mixtures of C_16:0_ and C_18:0_ fatty acids of known isotopic composition that were processed with each batch under identical conditions. Isotopic values obtained from modern references (Dataset S2) were corrected for the Suess Effect taking into consideration the year of collection (3).


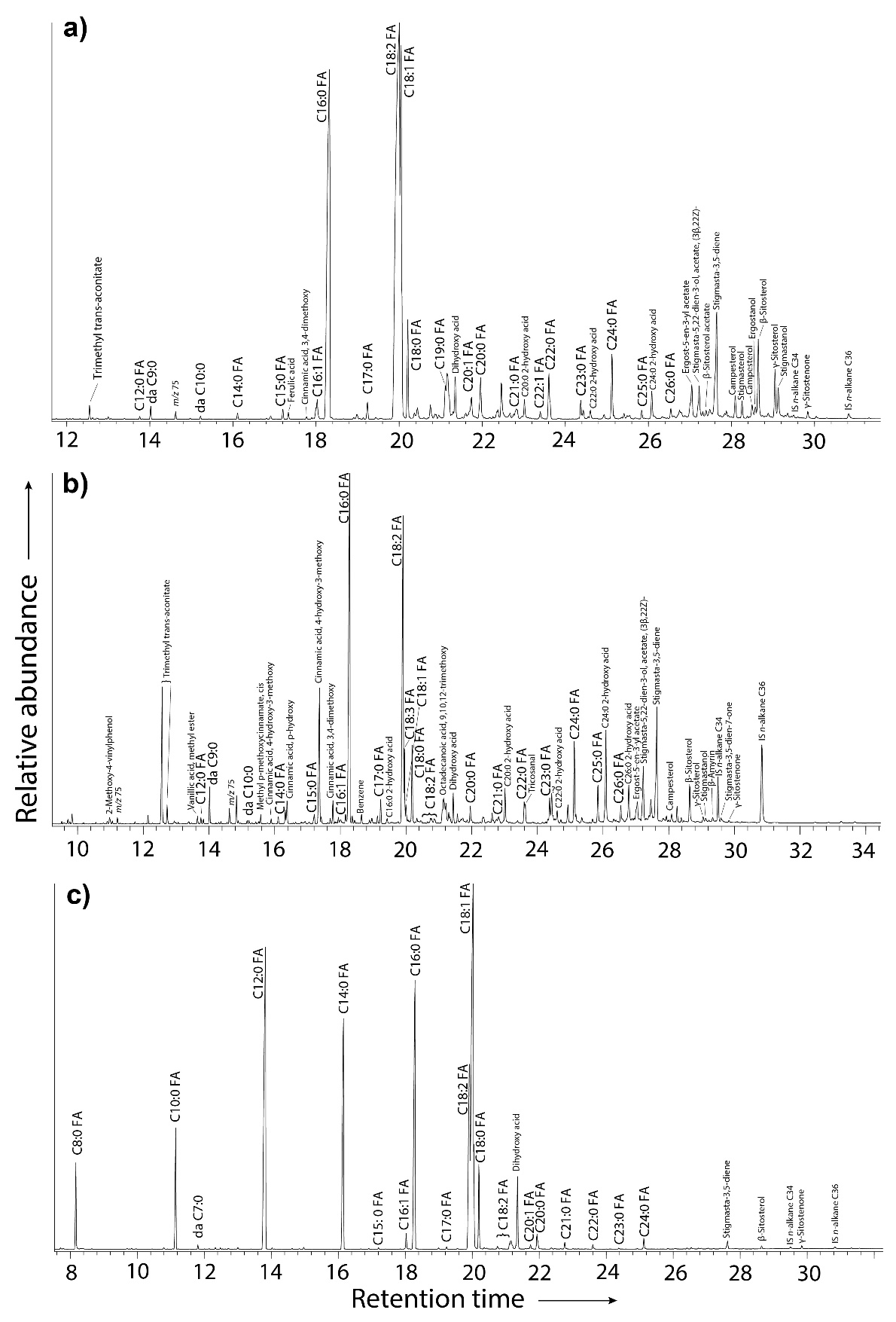


Fig. S1. a) a modern maize (*Zea mays*) kernel lipid extract from Brazilian Amazonia. Note the high abundance of unsaturated fatty acids C_18:2_ and C_18:1_; b) a modern maize cob lipid extract from Brazilian Amazonia; c) a modern lipid extract of palm nut (*Butia catarinensis*), from Santa Catarina, Brazil. Note the large C_12:0_ peak.

Table S1. Sample information

| **Phase** | **Sample I.D.** | **Sherd I.D.** | **Site** | **Site code** | **Region** | **width** | **length** | **thick- ness** | **Surface treatment** | **Sherd segment** |
| --- | --- | --- | --- | --- | --- | --- | --- | --- | --- | --- |
| Taquara-Itararé | BP-10018 |  | Bupeva II |  | Babitonga Bay | 22 | 28 | 7 | plain | body |
| Taquara-Itararé | BP-10027 |  | Bupeva II |  | Babitonga Bay | 34 | 51 | 7 | plain | body |
| Taquara-Itararé | BP-10028 |  | Bupeva II |  | Babitonga Bay | 39 | 46 | 9 | plain | body |
| Taquara-Itararé | BP-10029 |  | Bupeva II |  | Babitonga Bay | 28 | 34 | 6 | plain | body |
| Taquara-Itararé | BP-10043 |  | Bupeva II |  | Babitonga Bay | 26 | 37 | 9 | plain | body |
| Taquara-Itararé | BP-10044 |  | Bupeva II |  | Babitonga Bay | 25 | 34 | 11 | plain | body |
| Taquara-Itararé | BP-10045 |  | Bupeva II |  | Babitonga Bay | 32 | 31 | 9 | plain | body |
| Taquara-Itararé | BP-10092 |  | Bupeva II |  | Babitonga Bay | 25 | 25 | 5 | plain | rim |
| Taquara-Itararé | BP-10097 |  | Bupeva II |  | Babitonga Bay | 29 | 28 | 9 | plain | rim |
| Taquara-Itararé | BP-10100 |  | Bupeva II |  | Babitonga Bay | 24 | 38 | 7 | plain | body |
| Taquara-Itararé | BP-10101 |  | Bupeva II |  | Babitonga Bay | 28 | 35 | 8 | plain | body |
| Taquara-Itararé | BP-10108 |  | Bupeva II |  | Babitonga Bay | 39 | 46 | 5 | plain | rim |
| Taquara-Itararé | BP-10109 |  | Bupeva II |  | Babitonga Bay | 41 | 49 | 8 | plain | body |
| Taquara-Itararé | BP-10110 |  | Bupeva II |  | Babitonga Bay | 34 | 41 | 7 | plain | body |
| Taquara-Itararé | BP-10111 |  | Bupeva II |  | Babitonga Bay | 27 | 37 | 7 | plain | body |
| Taquara-Itararé | BP-10112 |  | Bupeva II |  | Babitonga Bay | 38 | 22 | 7 | plain | body |
| Taquara-Itararé | BP-10113 |  | Bupeva II |  | Babitonga Bay | 25 | 30 | 7 | plain | rim |
| Taquara-Itararé | BP-9712 |  | Bupeva II |  | Babitonga Bay | 26 | 31 | 9 | plain | rim |
| Taquara-Itararé | BP-9721 |  | Bupeva II |  | Babitonga Bay | 26 | 34 | 7 | plain | body |
| Taquara-Itararé | BP-9768 |  | Bupeva II |  | Babitonga Bay | 30 | 40 | 7 | plain | rim |
| Taquara-Itararé | BP-9813 |  | Bupeva II |  | Babitonga Bay | 44 | 48 | 7 | plain | body |
| Taquara-Itararé | BP-9821 |  | Bupeva II |  | Babitonga Bay | 29 | 42 | 9 | plain | body |
| Taquara-Itararé | BP-9823 |  | Bupeva II |  | Babitonga Bay | 27 | 41 | 9 | plain | body |
| Taquara-Itararé | BP-9824 |  | Bupeva II |  | Babitonga Bay | 31 | 36 | 9 | plain | rim |
| Taquara-Itararé | BP-9855 |  | Bupeva II |  | Babitonga Bay | 29 | 26 | 8 | plain | body |
| Taquara-Itararé | BP-9891 |  | Bupeva II |  | Babitonga Bay | 28 | 36 | 6 | plain | body |
| Taquara-Itararé | BP-9892 |  | Bupeva II |  | Babitonga Bay | 38 | 50 | 8 | plain | rim |
| Taquara-Itararé | BP-9914 |  | Bupeva II |  | Babitonga Bay | 28 | 49 | 10 | plain | rim |
| Taquara-Itararé | BP-9918 |  | Bupeva II |  | Babitonga Bay | 48 | 70 | 9 | plain | body |
| Taquara-Itararé | BP-9920 |  | Bupeva II |  | Babitonga Bay | 36 | 35 | 9 | plain | body |
| Taquara-Itararé | BP-9924 |  | Bupeva II |  | Babitonga Bay | 23 | 29 | 8 | plain | body |
| Taquara-Itararé | BP-9969 |  | Bupeva II |  | Babitonga Bay | 23 | 34 | 8 | plain | rim |
| Taquara-Itararé | BP-9990 |  | Bupeva II |  | Babitonga Bay | 37 | 35 | 8 | plain | rim |
| Taquara-Itararé | BP-9993 |  | Bupeva II |  | Babitonga Bay | 24 | 26 | 10 | plain | rim |
| Taquara-Itararé | IT-1206 |  | Itacoara |  | Babitonga Bay | 33 | 36 | 8 | plain | body |
| Taquara-Itararé | IT-1208 | 804 | Itacoara |  | Babitonga Bay | 37 | 38 | 9 | plain | rim |
| Taquara-Itararé | IT-1282 | 707 | Itacoara |  | Babitonga Bay | 36 | 44 | 7 | plain | body |
| Taquara-Itararé | IT-8324- 8387 | 802 | Itacoara |  | Babitonga Bay | 40 | 35 | 7 | plain | rim |
| Taquara-Itararé | IT-8356 | 852 | Itacoara |  | Babitonga Bay | 26 | 35 | 6 | plain | body |
| Taquara-Itararé | IT-8374 |  | Itacoara |  | Babitonga Bay | 28 | 26 | 7 | plain | body |
| Taquara-Itararé | IT-8381 | 867 | Itacoara |  | Babitonga Bay | 22 | 22 | 6 | plain | body |
| Taquara-Itararé | IT-8382 | 839 | Itacoara |  | Babitonga Bay | 22 | 30 | 5 | plain | body |
| Taquara-Itararé | IT-8430 |  | Itacoara |  | Babitonga Bay | 39 | 64 | 6 | plain | rim |
| Taquara-Itararé | IT-8490 | 793 | Itacoara |  | Babitonga Bay | 40 | 24 | 6 | plain | rim |
| Taquara-Itararé | IT-8508 | 879 | Itacoara |  | Babitonga Bay | 21 | 23 | 4 | plain | body |
| Taquara-Itararé | IT-8531 | 886 | Itacoara |  | Babitonga Bay | 20 | 27 | 6 | plain | body |
| Taquara-Itararé | IT-8614 |  | Itacoara |  | Babitonga Bay | 30 | 27 | 4 | plain | body |
| Taquara-Itararé | IT-8617 | 909 | Itacoara |  | Babitonga Bay | 55 | 48 | 6 | plain | body |
| Taquara-Itararé | IT-8618 | 803 | Itacoara |  | Babitonga Bay | 35 | 40 | 5 | plain | rim |
| Taquara-Itararé | IT-9480 | 921 | Itacoara |  | Babitonga Bay | 39 | 38 | 8 | plain | body |
| Taquara-Itararé | PIN-383 | 1888 | Pinheiro 8 |  | Babitonga Bay | 64 | 63 | 9 | plain | body |
| Taquara-Itararé | PIN-384A |  | Pinheiro 8 |  | Babitonga Bay | 59 | 92 | 6 | plain | rim |
| Taquara-Itararé | PIN-384B |  | Pinheiro 8 |  | Babitonga Bay | 50 | 105 | 7 | plain | body |
| Taquara-Itararé | PIN-385A | 1889 | Pinheiro 8 |  | Babitonga Bay | 71 | 74 | 8 | plain | body |
| Taquara-Itararé | PIN-385B | 1886 | Pinheiro 8 |  | Babitonga Bay | 38 | 61 | 6 | plain | body |
| Taquara-Itararé | G_16P |  | Galheta-IV |  | Laguna |  |  |  |  |  |
| Taquara-Itararé | G_17P |  | Galheta-IV |  | Laguna |  |  |  |  |  |
| Taquara-Itararé | G_18E |  | Galheta-IV |  | Laguna |  |  |  |  |  |
| Taquara-Itararé | G_18P |  | Galheta-IV |  | Laguna |  |  |  |  |  |
| Taquara-Itararé | G_19 |  | Galheta-IV |  | Laguna |  |  |  |  |  |
| Taquara-Itararé | G_20P |  | Galheta-IV |  | Laguna |  |  |  |  |  |
| Taquara-Itararé | G_21P |  | Galheta-IV |  | Laguna |  |  |  |  |  |
| Taquara-Itararé | G_22P |  | Galheta-IV |  | Laguna |  |  |  |  |  |
| Taquara-Itararé | G_24 |  | Galheta-IV |  | Laguna |  |  |  |  |  |
| Taquara-Itararé | G_25 |  | Galheta-IV |  | Laguna |  |  |  |  |  |
| Taquara-Itararé | G_26E |  | Galheta-IV |  | Laguna |  |  |  |  |  |
| Taquara-Itararé | G_26P |  | Galheta-IV |  | Laguna |  |  |  |  |  |
| Taquara-Itararé | G_27P |  | Galheta-IV |  | Laguna |  |  |  |  |  |
| Taquara-Itararé | G_CRUST |  | Galheta-IV |  | Laguna |  |  |  |  |  |
| Taquara-Itararé | G_sol |  | Galheta-IV |  | Laguna |  |  |  |  |  |
| Guarani | IT-3214 |  | Itacoara |  | Babitonga Bay | 26 | 25 | 7 | plain | body |
| Guarani | IT-5793 | 11 | Itacoara |  | Babitonga Bay | 34 | 92 | 10 | corrugated | body |
| Guarani | IT-699 | 699 | Itacoara |  | Babitonga Bay | 40 | 45 | 6 | plain | body |
| Guarani | IT-8326 | 716 | Itacoara |  | Babitonga Bay | 23 | 29 | 8 | corrugated | body |
| Guarani | IT-8433 |  | Itacoara |  | Babitonga Bay | 29 | 30 | 13 | plain | rim |
| Guarani | IT-8518 | 711 | Itacoara |  | Babitonga Bay | 26 | 29 | 8 | corrugated | body |
| Guarani | IT-9140 |  | Itacoara |  | Babitonga Bay | 19 | 42 | 12 | plain | body |
| Guarani | IT-9482+8499 | 708 | Itacoara |  | Babitonga Bay | 27 | 65 | 7 | corrugated | body |
| Guarani | PG-502 | 3206 | Poço Grande |  | Babitonga Bay | 28 | 35 | 8 | plain | body |
| Guarani | PG-503 | 10118 | Poço Grande |  | Babitonga Bay | 32 | 36 | 13 | plain | body |
| Guarani | PG-505 | 10122 | Poço Grande |  | Babitonga Bay | 28 | 36 | 11 | plain | body |
| Guarani | PG-506 | 3117 | Poço Grande |  | Babitonga Bay | 16 | 19 | 5 | plain | body |
| Guarani | PG-509 | 3107 | Poço Grande |  | Babitonga Bay | 14 | 27 | 10 | plain | body |
| Guarani | PG-510 | 10126 | Poço Grande |  | Babitonga Bay | 21 | 35 | 5 | plain | body |
| Guarani | PG-511e | 10137 | Poço Grande |  | Babitonga Bay | 18 | 22 | 6 | plain | body |
| Guarani | PG-516e | 3189 | Poço Grande |  | Babitonga Bay | 15 | 19 | 8 | plain | body |
| Guarani | PG-517e | 3167 | Poço Grande |  | Babitonga Bay | 17 | 31 | 7 | plain | body |
| Guarani | PG-521 | 3130 | Poço Grande |  | Babitonga Bay | 17 | 21 | 6 | plain | body |
| Guarani | PG-562 | 10119 | Poço Grande |  | Babitonga Bay | 22 | 31 | 11 | red slip | body |
| Guarani | PG-563 | 10125 | Poço Grande |  | Babitonga Bay | 27 | 34 | 9 | plain | rim |
| Guarani | PG-564 | 10139 | Poço Grande |  | Babitonga Bay | 23 | 38 | 7 | plain | body |
| Guarani | PG-565 | 10124 | Poço Grande |  | Babitonga Bay | 20 | 41 | 9 | plain | body |
| Guarani | PG-567 | 10117 | Poço Grande |  | Babitonga Bay | 22 | 31 | 9 | plain | body |
| Guarani | PG-568 | 10136 | Poço Grande |  | Babitonga Bay | 19 | 30 | 8 | plain | body |
| Guarani | PG-569 | 10121 | Poço Grande |  | Babitonga Bay | 22 | 28 | 8 | plain | body |
| Guarani | PG-570 | 3201 | Poço Grande |  | Babitonga Bay | 14 | 17 | 6 | plain | body |
| Guarani | PG-571 | 3202 | Poço Grande |  | Babitonga Bay | 12 | 21 | 5 | plain | body |
| Guarani | PG-572 | 10135 | Poço Grande |  | Babitonga Bay | 20 | 26 | 6 | plain | body |
| Guarani | PG-573e | 10133 | Poço Grande |  | Babitonga Bay | 16 | 25 | 10 | plain | body |
| Guarani | AR-01 |  | Aldeia Ribanceira 1 |  | Laguna | 26 | 52 | 14 | corrugated | body |
| Guarani | AR-02 |  | Aldeia Ribanceira 1 |  | Laguna | 26 | 52 | 14 | plain | body |
| Guarani | AR-03 |  | Aldeia Ribanceira 1 |  | Laguna | 33 | 47 | 11 | corrugated | body |
| Guarani | AR-04 |  | Aldeia Ribanceira 1 |  | Laguna | 36 | 43 | 9 | plain | body |
| Guarani | AR-05 |  | Aldeia Ribanceira 1 |  | Laguna | 24 | 47 | 13 | plain | body |
| Guarani | AR-06 |  | Aldeia Ribanceira 1 |  | Laguna | 40 | 54 | 11 | corrugated | body |
| Guarani | AR-07 |  | Aldeia Ribanceira 1 |  | Laguna | 33 | 43 | 11 | plain | body |
| Guarani | AR-08 |  | Aldeia Ribanceira 1 |  | Laguna | 37 | 52 | 10 | plain | body |
| Guarani | AR-09 |  | Aldeia Ribanceira 1 |  | Laguna | 33 | 61 | 8 | corrugated | body |
| Guarani | AR-10 |  | Aldeia Ribanceira 1 |  | Laguna | 30 | 42 | 5 | plain | body |
| Guarani | AR-11 |  | Aldeia Ribanceira 1 |  | Laguna | 35 | 65 | 13 | corrugated | body |
| Guarani | AR-12 |  | Aldeia Ribanceira 1 |  | Laguna | 67 | 80 | 11 | plain | body |
| Guarani | AR-13 |  | Aldeia Ribanceira 1 |  | Laguna | 49 | 66 | 17 | Painted | rim |
| Guarani | AR-14 |  | Aldeia Ribanceira 1 |  | Laguna | 55 | 75 | 16 | corrugated | body |
| Guarani | AR-15 |  | Aldeia Ribanceira 1 |  | Laguna | 66 | 80 | 16 | corrugated | body |
| Guarani | MA-A01 |  | Mato Alto 1 | SCMA1 | Laguna | 33 | 43 | 9 | corrugated | body |
| Guarani | MA-A04 |  | Mato Alto 1 | SCMA1 | Laguna | 32 | 35 | 15 | corrugated | body |
| Guarani | MA-A06 |  | Mato Alto 1 | SCMA1 | Laguna | 63 | 81 | 14 | corrugated | rim |
| Guarani | MA-A07 |  | Mato Alto 1 | SCMA1 | Laguna | 52 | 67 | 9 | corrugated | body |
| Guarani | MA-A08 |  | Mato Alto 1 | SCMA1 | Laguna | 43 | 60 | 12 | corrugated | body |
| Guarani | MA-A09 |  | Mato Alto 1 | SCMA1 | Laguna | 34 | 39 | 8 | corrugated | body |
| Guarani | MA-A10B |  | Mato Alto 1 | SCMA1 | Laguna | 46 | 75 | 12 | corrugated | body |
| Guarani | MA-A12 |  | Mato Alto 1 | SCMA1 | Laguna | 34 | 59 | 19 | painted | rim |
| Guarani | MA-A14 |  | Mato Alto 1 | SCMA1 | Laguna | 45 | 65 | 9 | corrugated | body |
| Guarani | MA-A16 |  | Mato Alto 1 | SCMA1 | Laguna | 53 | 73 | 17 | corrugated | rim |
| Guarani | MA-A17 |  | Mato Alto 1 | SCMA1 | Laguna | 69 | 153 | 15 | corrugated | rim |
| Guarani | MA-A18 |  | Mato Alto 1 | SCMA1 | Laguna | 73 | 109 | 11 | corrugated | rim |
| Guarani | MA-A23 |  | Mato Alto 1 | SCMA1 | Laguna | 38 | 54 | 5 | irregular marked | rim |
| Guarani | MA-A24 |  | Mato Alto 1 | SCMA1 | Laguna | 57 | 69 | 15 | plain | body |
| Guarani | MA-A27 |  | Mato Alto 1 | SCMA1 | Laguna | 44 | 45 | 14 | fingernail marked | body |
| Guarani | MA-A28 |  | Mato Alto 1 | SCMA1 | Laguna | 37 | 63 | 9 | painted | rim |
| Guarani | MA-A30 |  | Mato Alto 1 | SCMA1 | Laguna | 38 | 42 | 7 | fingernail marked | body |
| Guarani | MA-A31 |  | Mato Alto 1 | SCMA1 | Laguna | 29 | 33 | 8 | fingernail marked | body |
| Guarani | MA-A34 |  | Mato Alto 1 | SCMA1 | Laguna | 45 | 76 | 7 | plain | body |
| Guarani | MA-A36 |  | Mato Alto 1 | SCMA1 | Laguna | 37 | 60 | 12 | corrugated | body |
| Guarani | RDF2-606 |  | Riacho dos Franciscos 2 |  | Laguna | 43 | 55 | 8 | Painted | body |
| Guarani | RDF2-639 |  | Riacho dos Franciscos 2 |  | Laguna | 68 | 58 | 10 | corrugated | rim |
| Guarani | RDF2-641 |  | Riacho dos Franciscos 2 |  | Laguna | 40 | 75 | 15 | corrugated | body |
| Guarani | RDF2-642 |  | Riacho dos Franciscos 2 |  | Laguna | 55 | 80 | 7 | plain | rim |
| Guarani | RDF2-670 |  | Riacho dos Franciscos 2 |  | Laguna | 40 | 50 | 6 | corrugated | body |
| Guarani | RDF2-686 |  | Riacho dos Franciscos 2 |  | Laguna | 57 | 65 | 9 | corrugated | rim |
| Guarani | RDF2-691 |  | Riacho dos Franciscos 2 |  | Laguna | 22 | 28 | 7 | plain | rim |
| Guarani | RDF2-693 |  | Riacho dos Franciscos 2 |  | Laguna | 64 | 45 | 11 | plain | body |
| Guarani | RDF2-732 |  | Riacho dos Franciscos 2 |  | Laguna | 40 | 34 | 8 | corrugated | rim |
| Guarani | RDF2-750 |  | Riacho dos Franciscos 2 |  | Laguna | 79 | 66 | 17 | plain | body |
| Guarani | RDF2-768 |  | Riacho dos Franciscos 2 |  | Laguna | 40 | 67 | 7 | irregular marked | rim |
| Guarani | RDF2-796 |  | Riacho dos Franciscos 2 |  | Laguna | 65 | 110 | 9 | corrugated | rim |
| Guarani | RDF2-824 |  | Riacho dos Franciscos 2 |  | Laguna | 25 | 27 | 8 | plain | rim |
| Guarani | RDF2-831 |  | Riacho dos Franciscos 2 |  | Laguna | 55 | 90 | 7 | Painted | rim |
| Guarani | RDF2-841 |  | Riacho dos Franciscos 2 |  | Laguna | 50 | 60 | 10 | corrugated | rim |
| Guarani | RDF2-857 |  | Riacho dos Franciscos 2 |  | Laguna | 62 | 48 | 13 | plain | rim |
| Guarani | RDF2-859 |  | Riacho dos Franciscos 2 |  | Laguna | 72 | 53 | 13 | corrugated | rim |
| Guarani | RDF2-865 |  | Riacho dos Franciscos 2 |  | Laguna | 66 | 59 | 10 | irregular marked | rim |
| Guarani | RDF2-907 |  | Riacho dos Franciscos 2 |  | Laguna | 48 | 42 | 7 | irregular marked | rim |
| Guarani | RDF2-930 |  | Riacho dos Franciscos 2 |  | Laguna | 52 | 41 | 9 | plain | rim |
| Guarani | SCLGN50-174 |  | Laguna 50 | SCLGN-050 | Laguna | 70 | 122 | 18 | plain | body |
| Guarani | SCLGN50-175 |  | Laguna 50 | SCLGN-050 | Laguna | 94 | 90 | 8 | plain | body |
| Guarani | SCLGN50-176 |  | Laguna 50 | SCLGN-050 | Laguna | 73 | 67 | 9 | plain | body |
| Guarani | SCLGN50-177 |  | Laguna 50 | SCLGN-050 | Laguna | 55 | 56 | 11 | plain | rim |
| Guarani | SCLGN50-178 |  | Laguna 50 | SCLGN-050 | Laguna | 62 | 86 | 9 | corrugated | rim |
| Guarani | SCLGN50-179 |  | Laguna 50 | SCLGN-050 | Laguna | 69 | 61 | 13 | plain | body |
| Guarani | SCLGN50-180 |  | Laguna 50 | SCLGN-050 | Laguna | 33 | 38 | 8 | corrugated | rim |
| Guarani | SCLGN50-181 |  | Laguna 50 | SCLGN-050 | Laguna | 25 | 34 | 8 | corrugated | body |
| Guarani | SCLGN50-182 |  | Laguna 50 | SCLGN-050 | Laguna | 27 | 58 | 16 | plain | body |
| Guarani | SCLGN50-183 |  | Laguna 50 | SCLGN-050 | Laguna | 29 | 23 | 14 | plain | body |
| Guarani | SCLGN50-184 |  | Laguna 50 | SCLGN-050 | Laguna | 29 | 36 | 6 | plain | rim |
| Guarani | SCLGN50-185 |  | Laguna 50 | SCLGN-050 | Laguna | 56 | 54 | 20 | corrugated | body |
| Guarani | SCLGN50-186 |  | Laguna 50 | SCLGN-050 | Laguna | 30 | 52 | 16 | plain | body |
| Guarani | SCLGN50-187 |  | Laguna 50 | SCLGN-050 | Laguna | 47 | 58 | 22 | painting | body |
| Guarani | SCLGN50-188 |  | Laguna 50 | SCLGN-050 | Laguna | 41 | 43 | 14 | plain | rim |
| Guarani | SCLGN50-189 |  | Laguna 50 | SCLGN-050 | Laguna | 22 | 33 | 17 | plain | body |
| Guarani | SCLGN50-190 |  | Laguna 50 | SCLGN-050 | Laguna | 26 | 46 | 14 | plain | body |
| Guarani | SCLGN50-191 |  | Laguna 50 | SCLGN-050 | Laguna | 33 | 58 | 23 | plain | body |
| Guarani | SCLGN50-192 |  | Laguna 50 | SCLGN-050 | Laguna | 47 | 46 | 12 | plain | body |
| Guarani | SCLGN50-193 |  | Laguna 50 | SCLGN-050 | Laguna | 21 | 61 | 21 | slip lines | body |
| Guarani | SCLGN50-194 |  | Laguna 50 | SCLGN-050 | Laguna | 31 | 36 | 10 | plain | body |
| Guarani | SCLGN50-195 |  | Laguna 50 | SCLGN-050 | Laguna | 40 | 73 | 14 | corrugated | body |
| Guarani | SCLGN50-196 |  | Laguna 50 | SCLGN-050 | Laguna | 45 | 48 | 17 | plain | body |
| Guarani | SCLGN50-197 |  | Laguna 50 | SCLGN-050 | Laguna | 44 | 51 | 20 | plain | body/shoulder |
| Guarani | SCLGN50-198 |  | Laguna 50 | SCLGN-050 | Laguna | 39 | 41 | 6 | plain | rim |
| Guarani | SCLGN50-199 |  | Laguna 50 | SCLGN-050 | Laguna | 47 | 51 | 16 | plain | body |
| Guarani | SCLGN50-200 |  | Laguna 50 | SCLGN-050 | Laguna | 41 | 55 | 5 | plain | body |
| Guarani | SCLGN50-201 |  | Laguna 50 | SCLGN-050 | Laguna | 43 |  | 12 | plain | rim |
| Guarani | SCLGN50-202 |  | Laguna 50 | SCLGN-050 | Laguna | 52 | 75 | 11 | plain | body |
| Guarani | SCLGN50-203 |  | Laguna 50 | SCLGN-050 | Laguna | 31 | 45 | 20 | plain | body |
| Guarani | SCLGN50-204 |  | Laguna 50 | SCLGN-050 | Laguna | 41 | 41 | 11 | plain | body |
| Guarani | SCLGN50-205 |  | Laguna 50 | SCLGN-050 | Laguna | 29 | 36 | 11 | corrugated | rim |
| Guarani | SCLGN50-206 |  | Laguna 50 | SCLGN-050 | Laguna | 39 | 39 | 9 | corrugated | body |
| Guarani | SCLGN50-207 |  | Laguna 50 | SCLGN-050 | Laguna | 37 | 53 | 23 | plain | body |
| Guarani | SCLGN50-208 |  | Laguna 50 | SCLGN-050 | Laguna | 34 | 60 | 20 | plain | body |
| Guarani | SCLGN50-209 |  | Laguna 50 | SCLGN-050 | Laguna | 40 | 57 | 9 | plain | body |
| Guarani | SCLGN50-210 |  | Laguna 50 | SCLGN-050 | Laguna | 45 | 56 | 10 | plain | body |
| Guarani | SCLGN50-211 |  | Laguna 50 | SCLGN-050 | Laguna | 28 | 39 | 12 | plain | body |
| Guarani | SCLGN50-212 |  | Laguna 50 | SCLGN-050 | Laguna | 24 | 46 | 11 | plain | body |
| Guarani | SCLGN50-213 |  | Laguna 50 | SCLGN-050 | Laguna |  |  |  |  |  |
| Guarani | SCLGN50-214 |  | Laguna 50 | SCLGN-050 | Laguna | 51 | 65 | 19 | plain | body |
| Guarani | SCLGN50-215 |  | Laguna 50 | SCLGN-050 | Laguna | 30 | 44 | 8 | plain | body |
| Guarani | SCLGN50-216 |  | Laguna 50 | SCLGN-050 | Laguna | 17 | 39 | 12 | plain | body |
| Guarani | SCLGN50-218 |  | Laguna 50 | SCLGN-050 | Laguna | 27 | 33 | 15 | plain | body |
| Guarani | SCLGN50-273 |  | Laguna 50 | SCLGN-050 | Laguna |  |  |  |  |  |
| Guarani | CAM-11 | 51.2...11? | Camping | PS-02-Camping | Patos Lagoon | 42 | 46 | 13 | plain | body |
| Guarani | CAM-115 | 51.2.115 | Camping | PS-02-Camping | Patos Lagoon | 28 | 37 | 12 | corrugated | body |
| Guarani | CAM-158 | 51.2.158 | Camping | PS-02-Camping | Patos Lagoon | 34 | 51 | 14 | plain | body |
| Guarani | CAM-2 | 51.2.002 | Camping | PS-02-Camping | Patos Lagoon | 46 | 43 | 12 | corrugated | body |
| Guarani | CAM-29 | 51.2.029 | Camping | PS-02-Camping | Patos Lagoon | 32 | 44 | 15 | corrugated | body |
| Guarani | CAM-79 | 51.2.079 | Camping | PS-02-Camping | Patos Lagoon | 31 | 50 | 9 | corrugated | body |
| Guarani | TOT-004 | 66.3.4 | Totó | PS-03-Totó | Patos Lagoon | 45 | 48 | 13 | corrugated | body |
| Guarani | TOT-024 | 66.3.24 | Totó | PS-03-Totó | Patos Lagoon | 30 | 36 | 10 | plain | rim |
| Guarani | TOT-027 | 66.3.27 | Totó | PS-03-Totó | Patos Lagoon | 24 | 37 | 12 | corrugated | body |
| Guarani | TOT-035 | 66.3.35 | Totó | PS-03-Totó | Patos Lagoon | 27 | 34 | 11 | plain | body |
| Guarani | TOT-121 | 66.1.121 | Totó | PS-03-Totó | Patos Lagoon | 16 | 82 | 21 | plain | body |
| Guarani | TOT-160 | 66.3.160 | Totó | PS-03-Totó | Patos Lagoon | 37 | 43 | 10 | corrugated | body |
| Guarani | TOT-173 | 66.3.173 | Totó | PS-03-Totó | Patos Lagoon | 29 | 47 | 12 | plain | body |
| Guarani | TOT-192 | 66.3.192 | Totó | PS-03-Totó | Patos Lagoon | 24 | 31 | 9 | corrugated | body |
| Guarani | TOT-198 | 66.3.198 | Totó | PS-03-Totó | Patos Lagoon | 42 | 38 | 12 | plain | body |
| Guarani | TOT-246 | 70.03.246 | Totó | PS-03-Totó | Patos Lagoon | 33 | 61 | 9 | plain | rim |
| Guarani | TOT-247 | 70.03.247 | Totó | PS-03-Totó | Patos Lagoon | 28 | 71 | 8 | plain | rim |
| Guarani | TOT-256 | 70.03.256 | Totó | PS-03-Totó | Patos Lagoon | 37 | 41 | 12 | plain | rim |
| Guarani | TOT-278 | 70.03.278 | Totó | PS-03-Totó | Patos Lagoon | 40 | 52 | 20 | corrugated | body |
| Guarani | TOT-280 | 70.03.280 | Totó | PS-03-Totó | Patos Lagoon | 29 | 36 | 8 | plain | rim |
| Guarani | TOT-308e | 70.03.308 | Totó | PS-03-Totó | Patos Lagoon | 20 | 29 | 11 | plain | rim |
| Guarani | TOT-312 | 70.03.312 | Totó | PS-03-Totó | Patos Lagoon | 26 | 41 | 20 | plain | rim |
| Guarani | TOT-318 | 70.03.318 | Totó | PS-03-Totó | Patos Lagoon | 36 | 49 | 12 | corrugated | body |
| Guarani | TOT-428 | 66.3.428 | Totó | PS-03-Totó | Patos Lagoon | 28 | 40 | 8 | plain | body |

Table S2. Radiocarbon dates

| **Site, level** | **Material** | **Lab-ID** | **Radiocarbon Age (BP)** | **δ^13^C (‰)** | **Calibrated Age (SHCAL-20) (4)** |
| --- | --- | --- | --- | --- | --- |
| Riacho dos Franciscos (RFC1), catalogo 1.34.1, area 1, quadra 4, caixa 1 | Plant charcoal | LTL22559 | 770 ± 40 BP | -31.2 ± 0.8 | 730-565 cal BP |
| Riacho dos Franciscos (RFC2), catalogo 3.34.3.1, area 3, TR1 UN17, caixa 1 | Plant charcoal | LTL22560 | 461 ± 40 BP | -33.2 ± 0.2 | 534-327 cal BP |
| Aldeia Ribanceira, ARI - Nivel 7/Quadra 11-12N/Area 2 | Plant charcoal | LTL22562 | 502 ± 40 BP | -31.0 ± 0.4 | 549-341 cal BP |
| Aldeia Ribanceira, ARI - Nivel 6/Quadra 12W/Area 2 | Plant charcoal | LTL22563 | 404 ± 40 BP | -23.9 ± 0.6 | 498-322 cal BP |
| Aldeia Ribanceira, ARI - Nivel 5/Quadra UE Centara | Plant charcoal | LTL22564 | 802 ± 40 BP | -30.6 ± 0.5 | 762-571 cal BP |

**Table S3.** Mean carbon isotope and concentration values δ^13^C_16:0_, δ^13^C_18:0_ and δ^13^C_18:1_ for four lipid sources used for Bayesian modelling.

|  | **Maize** | **Marine** | **C3 plants** | **Ruminant** |
| --- | --- | --- | --- | --- |
| **δ^13^C_16:0_** | -15.4 | -21.4 | -28.1 | -29.8 |
| **δ^13^C_18:0_** | -15.5 | -21.5 | -27.5 | -31.6 |
| **δ^13^C_18:1_** | -12.7 | -22.3 | -26.6 | -31.1 |
| **C_16:0_ (% total FA)** | 13.7 ± 0.2 | 15.5 ± 0.6 | 10.2 ± 2.0 | 22.6 ± 1.3 |
| **C_18:0_ (% total FA)** | 1.9 ± 0 | 3.4 ± 0.1 | 3.2 ± 0.9 | 17.7 ± 0.8 |
| **C_18:1_ (% total FA)** | 24.4 ± 0.5 | 15.5 ± 0.5 | 36.4 ± 7.7 | 37.8 ± 2.3 |

**SI References**

1. Cramp, Evershed, Reconstructing aquatic resource exploitation in human prehistory using lipid biomarkers and stable isotopes. *Treatise on Geochemistry* **14**, 319–339 (2014).
2. O. E. Craig, et al., Distinguishing wild ruminant lipids by gas chromatography/combustion/isotope ratio mass spectrometry*. Rapid Commun. Mass Spectrom.* **26**, 2359–2364 (2012).
3. H. Hellevang, P. Aagaard, Constraints on natural global atmospheric CO2 fluxes from 1860 to 2010 using a simplified explicit forward model. *Sci. Rep*. **5**, 17352 (2015).
4. A. G. Hogg, et al., SHCal20 Southern Hemisphere Calibration, 0–55,000 Years cal BP. *Radiocarbon* **62**, 759–778 (2020).
